# The *Cuídalas* program: an AI-supported community-based approach to breast cancer screening in low-resource settings

**DOI:** 10.3389/fonc.2026.1794792

**Published:** 2026-04-10

**Authors:** Wolmark Xiques-Molina, Ivan David Lozada-Martinez, Ernesto Barceló-Martinez, Víctor Mario Noble-Ramos

**Affiliations:** 1 Research & Development Unit, Cure Latam Health Technologies, Barranquilla, Colombia; 2 Biomedical Scientometrics and Evidence-Based Research Unit, Department of Health Sciences, Universidad de la Costa, Barranquilla, Colombia; 3 Clínica Colsanitas S.A., Clínica Iberoamérica, Barranquilla, Colombia; 4 Department of Industrial Engineering, Universidad de Córdoba, Monteria, Colombia

**Keywords:** breast neoplasms, diagnostic screening programs, early detection of cancer, low-resource settings, primary health care

## Abstract

Breast cancer remains one of the leading causes of morbidity, mortality, and healthcare costs related to cancer worldwide. Despite advances in early diagnosis and treatment, mortality rates remain disproportionately high in low- and middle-income countries, due to the lack of access to timely and adequate screening programs and healthcare services. The aim of this manuscript is to describe the *Cuídalas* Program, an artificial intelligence-supported, community-based initiative designed to improve breast cancer screening and follow-up in low-resource areas of Colombia. Rather than constituting an experimental or hypothesis-driven investigation, this report presents aggregated operational indicators derived from routine program implementation between March 2023 and September 2024. During this period, community outreach and mobile diagnostic strategies facilitated access to screening services for 54,970 women across multiple regions. Among those screened, aggregated indicators documented suspicious findings requiring further evaluation and confirmed cancer diagnoses through established referral pathways. Complementary education initiatives engaged 291,330 individuals, reinforcing awareness and early-detection literacy. This program introduces an innovative four-phase strategy: 1) Demand induction supported by artificial intelligence, 2) Organized population screening with portable devices supported by artificial intelligence, 3) Follow-up of the comprehensive care pathway, and 4) Surveillance and notification protocol. The program encompasses three key approaches: 1) Preventive; 2) Predictive; and 3) Resolutive. The *Cuídalas* Program aims to reduce mortality from late-stage breast cancer diagnoses, decrease potentially avoidable early mortality from breast cancer, lower medical costs, and reduce the need for aggressive treatments.

## 1 Introduction

Breast cancer remains one of the leading causes of morbidity, mortality, and healthcare costs related to cancer worldwide (1). Recent estimates from the Global Cancer Observatory (GLOBOCAN) indicate that approximately 2.3 million cases were diagnosed in 2022, making it the second most common cancer globally, the most frequent cancer among women, and the fourth deadliest overall (2).

Despite advances in early diagnosis and treatment, mortality rates remain disproportionately high in low- and middle-income countries (3), due to the lack of access to timely and adequate screening programs and healthcare services (4). This results in a high proportion of late-stage cases and low survival rates. Although clinical breast exams and screening mammography have been proposed as strategies for screening at-risk women, they require specific technical and operational criteria for implementation (5, 6). The absence of trained healthcare personnel to perform adequate clinical breast exams, the limited availability of hospital infrastructure, and the lack of access to rural or geographically dispersed populations are widely discussed barriers addressed by global initiatives, hindering the implementation of traditional strategies (7, 8).

The use of emerging technologies applied to community settings could serve as an innovative and replicable tool in low-resource environments, especially where traditional strategies cannot be implemented (9). Previously, some portable devices based on elastography and supported by artificial intelligence (AI) have been developed, showing utility, applicability, and scalability in low-resource settings (10, 11). However, a comprehensive program is needed to ensure adherence to the pathway for early detection and timely treatment of clinically relevant breast lesions or breast cancer.

This article examines the social, geographic, and systemic barriers to breast cancer screening in low-resource settings, with particular attention to the Colombian context. It further describes the conceptual design, implementation process, and preliminary operational results of the *Cuídalas* Program, a community-based, AI-supported model developed to address these challenges and improve access to early detection.

This manuscript reports aggregated, de-identified operational indicators from the implementation of a routine, community-based screening and care-navigation program. The report is intended to describe feasibility, workflow, and program reach rather than to evaluate clinical effectiveness or comparative outcomes. No experimental intervention was assigned, and no identifiable individual-level research dataset was created for this publication. Therefore, according to institutional policy and applicable regulations for program evaluation/quality-improvement reporting, formal ethics committee approval was not required.

This manuscript should be interpreted as a narrative description and implementation analysis of a community-based program rather than as an original clinical research study.

## 2 Social determinants of health in breast cancer in Colombia

The state of social determinants of health in a given region has been described as an essential and determining factor for the efficacy and efficiency of breast cancer screening and control techniques (12, 13). For this reason, low-resource settings present a significant challenge, as these determinants are often vulnerable, hindering the implementation and adherence to comprehensive pathways for the timely detection of breast cancer (14). Moreover, the vulnerability of social determinants of health is itself a risk factor for breast cancer mortality, even after early detection, due to the lack of a robust network of healthcare services and social support (14, 15).

This absence prevents timely treatment and adequate rehabilitation. Socially isolated individuals lack adequate instrumental support, face high out-of-pocket or catastrophic expenses, and often do not receive psychological, financial, or employment-related assistance, creating a mechanism that contributes to an unfavorable prognosis (16).

Social determinants of health have been correlated with cancer risk, stage, and survival, and represent a valuable variable for the development of screening strategies, comprehensive care pathways, and the application of tools for the early detection and treatment of breast cancer (12–14). In Latin American and African countries, where there is a significant number of low- and middle-income nations, barriers and opportunities for the effective management of breast cancer have been described (17, 18). One of the most important limitations recognized is the failure of decision-makers to acknowledge this variable when establishing programs (17, 18).

Although evidence supports that traditional tools such as mammography, ultrasound, and clinical breast exams can positively impact the incidence of early-stage breast cancer cases, these results have been primarily obtained in high-income countries, where adherence to comprehensive breast cancer management pathways is ensured (19, 20).

According to the Gini index, Colombia is one of the most inequitable countries in the world (54.8 value) (21). In Latin America, Colombia stands out as one of the countries with the highest poverty rates, with 6.50% of the population experiencing multidimensional poverty (22). In this region, following Brazil, Mexico, and Argentina, Colombia ranks fourth in terms of the highest incidence-to-mortality ratio for absolute deaths (3,607 deaths in 2022) (2). According to data from Colombia’s National Cancer Institute, breast cancer has historically been one of the most common cancers, accounting for 18% of all cancer cases (23). Among all women who died, 49% were aged between 45 and 59 years, 56% had only a primary education, and 64% belonged to low or very low socioeconomic levels (23).

National statistics estimate that 52% of breast cancer cases detected in Colombia are in advanced stages (24). In 244 municipalities (22%) across the country, there are no breast health services available, and in 55% of municipalities, there is only one public hospital (25). Additionally, there are approximately 1.84 general practitioners per 1,000 inhabitants, with 61% of these practitioners concentrated in six states (26), highlighting a significant gap in coverage and access to clinical breast exams by adequately trained personnel or to infrastructure for accessing active screening strategies based on mammography or ultrasound.

In this country, national data indicate that 33% of the population lives in poverty, and 31.50% are in a state of vulnerability (27). Additionally, out of the 1,103 municipalities, 368 and 261 are classified as rural and sparsely populated rural municipalities, respectively (57%) (27). This highlights the challenge of applying traditional strategies that were not originally designed for this context and require a robust infrastructure and a coordinated model designed to expand equitable and timely access to screening services for women at risk or symptomatic, who need evaluation for clinically relevant breast lesions or breast cancer.

In this situation, a primary care program is needed that provides tools to effectively address these barriers and screen the largest proportion of at-risk women, particularly those who cannot access mammography, ultrasound, or clinical breast exams performed by trained personnel.

## 3 *Cuídalas* program: description and preliminary results

This section provides a descriptive account of the program’s structure, implementation phases, and preliminary operational indicators derived from its early deployment. The results presented here are intended to illustrate feasibility and scalability, rather than clinical effectiveness or comparative outcomes.

To contextualize the operational reach of the *Cuídalas* Program, it is important to note that national mammography coverage in Colombia remains suboptimal. Recent estimates indicate that only about 31% of eligible women were screened in 2022 (28), while the 2015 Demographic and Health Survey reported a 48.10% participation rate among women aged 40–69 years (29). In Bogotá, a more recent analysis found that 54% of women aged 50–69 years had undergone mammography within the previous two years (30). Notably, the International Agency for Research on Cancer (IARC) currently reports the absence of complete national quantitative data on breast cancer screening coverage for Colombia (31). These figures, although methodologically heterogeneous, underscore the relevance of community-based initiatives such as *Cuídalas* to expand access and equity in breast health services across low-resource settings.

Recognizing the geographic, cultural, operational, and human barriers to implementing traditional breast cancer screening strategies in Colombia, along with the conceptual foundations of the Breast Health Global Initiative (BHGI) (20), previous experiences with programs developed in low-resource settings, and the World Health Organization’s (WHO) recommendations on breast cancer inequities, the *Cuídalas* Program was designed (32). This is a community-based primary care program supported by a large-scale demand generation strategy and a portable device powered by artificial intelligence, aimed at enhancing accessibility, timeliness, follow-up, and the early detection of clinically relevant breast lesions and breast cancer.

The *Cuídalas* Program embodies the core principles of community-engaged research: building trust through continuous presence in the community, culturally tailoring health communication, and leveraging the local nursing workforce as agents of change. Nurses acted as cultural brokers and advocates for women’s health, facilitating equitable access to early detection services and bridging the gap between innovative technology and vulnerable populations. These elements position the program not only as a technological innovation but also as a model of nursing-led community empowerment.

The *Cuídalas* Program operated within the framework of the existing Colombian healthcare system. Screening activities were conducted as part of community health services, and participants who required additional diagnostic evaluation or treatment were referred through standard healthcare pathways. Subsequent care, including imaging, biopsy, or oncological management when indicated, was provided through the participants’ regular health insurance coverage and affiliated provider networks. The program itself did not establish a separate insurance policy, as it functioned as a screening and care-navigation initiative integrated into routine health services.

Complementing current breast cancer screening guidelines in Colombia (33, 34) and Latin America (35), based on clinical practice guidelines, which establish that screening is conducted in asymptomatic populations, early diagnosis of the disease can eventually be achieved through traditional strategies, specifically, clinical breast exams or ultrasounds for women between 40 and 49 years of age, and annual mammograms for women between 50 and 69 years. The *Cuídalas* Program introduces an innovative four-phase strategy for the timely detection of clinically relevant breast lesions, ensuring adherence to the comprehensive pathway for access to curative therapies for breast neoplasms:

### 3.1 Phase 1: demand induction supported by AI

Using proprietary software (CureBot, API-Cure, and CURE Connect), along with instant messaging applications like WhatsApp, a direct contact mechanism is established, reaching up to 10,000 women within four hours. Phone numbers and mailing addresses are provided by the health insurance company. Scheduling is done online in real time through text messaging, mobile applications and by phone. The bot-assisted service automatically schedules appointments for women who consent to the scheduling. In the case of people without the capacity to receive digital scheduling or illiterate, priority is given to telephone contact. In this situation, for confirmed cases, direct communication is conducted through a trained human assistant, who assesses the level of breast cancer awareness and education based on sociodemographic, educational, cultural, health, geographic, and economic characteristics. The mass messaging system communicates the appointment request and confirmation across all available communication channels. This approach strengthens adherence to the screening schedule.

### 3.2 Phase 2: organized population screening

In public or private institutions, through voluntary or organized agreements, or at the woman’s residence, trained healthcare personnel, comprising nurses, nursing assistants, or community health workers, comprehensive screening is performed. Through training on population characterization and risk classification, as well as how to perform a visual inspection of the breast, health care personnel perform this process. This process can be performed with several novel portable tools with the potential to assess the structural integrity of the breast. In this experience, the *iBreastExam™* device was used.

This portable and scalable device, based on elastography and supported by AI and machine learning, automatically interprets and reproduces the results upon completion of the exam, which are then sent to both the health insurance company and the patient. The *iBreastExam™* measures the elastic model between normal and abnormal breast tissue, based on differences in tissue stiffness. Each breast contains 16 regions to be analyzed, with their size varying according to breast size. Scanning each region takes three seconds, and after two seconds, the device automatically calibrates, processing the information through the AI algorithm.

The iBreastExam™ is a handheld breast assessment device developed by UE LifeSciences and previously described in the scientific literature as a portable tool designed for use in low-resource settings (10, 11). In the *Cuídalas* Program, the device was implemented as part of a community-based screening strategy under routine health service conditions.

The activities described in this manuscript correspond to routine community-based screening and care-navigation services integrated within the Colombian healthcare system. This report presents aggregated operational indicators from program implementation and does not constitute a human-subject research study. All women provided written informed consent prior to screening as part of standard clinical practice. No identifiable individual-level data were analyzed for research purposes.

### 3.3 Phase 3: follow-up of the comprehensive care pathway

Once a suspicious or positive finding is detected during screening, the patient is referred to higher-level healthcare services for additional tests, such as ultrasound or mammography, and for definitive diagnosis through histopathology, if necessary. In cases where breast cancer is diagnosed, direct and intensive contact is made with health insurers and partner institutions to establish a timely plan for specialized care and treatment.

As part of the care-navigation strategy, telecommunication tools were used to support follow-up, coordination of diagnostic appointments, and referral tracking. This component facilitated communication between community teams, healthcare providers, and participants, particularly in geographically dispersed areas. Teleconsultation was used for coordination and guidance purposes and was not evaluated as a standalone intervention.

### 3.4 Phase 4: surveillance and notification protocol

In cases where suspicious findings are detected, but breast cancer is not confirmed, strict follow-up is conducted with personalized screening. For positive cases, a rigorous communication plan is implemented to continuously assess the patient’s social determinants of health, providing support in collaboration with the health insurance company throughout the woman’s rehabilitation and palliative care, until the final outcome.

The program consists of eight steps to complete the described phases: A) Induced demand; B) Education through digital campaigns; C) Population characterization; D) Risk classification; E) Visual inspection; F) Digital clinical examination using portable device supported by artificial intelligence; G) Referral to specialized services based on findings; and H) Follow-up with complementary services, ensuring compliance with the comprehensive care pathway for breast cancer in Colombia.

**Figure 1:**
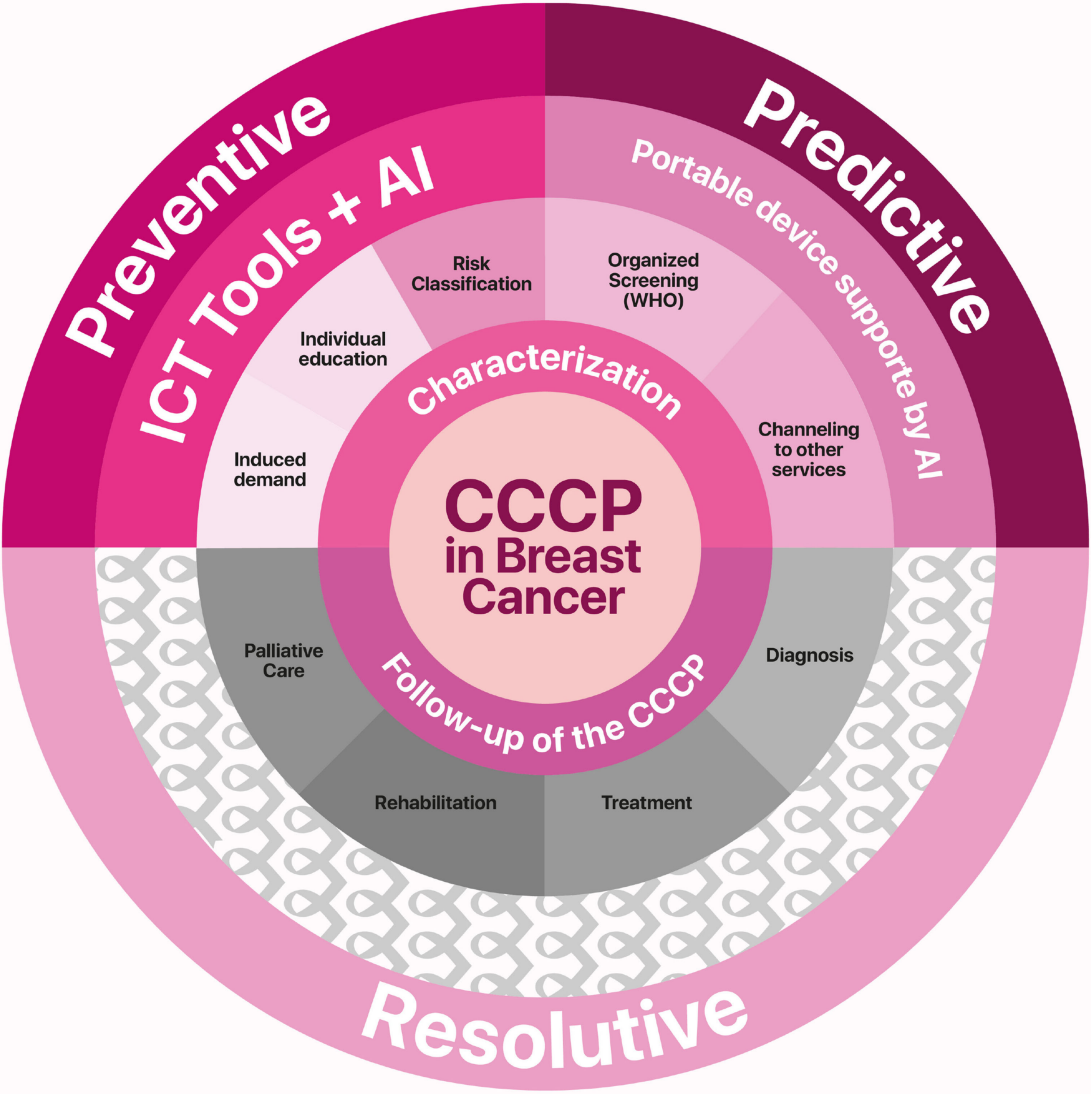
Three complementary approaches of the *Cuídalas* Program, Preventive, Predictive, and Care-Oriented, integrated within a community-based nursing model for breast health and early detection. AI, Artificial Intelligence; CCCP, Comprehensive Cancer Care Pathway; ICT, Information and Communication Technology; Source, authors.

In this way, the program encompasses three key approaches: 1) Preventive; 2) Predictive; and 3) Resolutive (**Figure 1**). From the preventive approach, with the support of information and communication technologies and artificial intelligence, it is possible to implement demand generation, provide individualized education on health and disease processes, and classify personalized risk. In the predictive approach, using a portable and scalable device, organized screening is conducted, and patients are referred to other healthcare services to confirm or rule out a diagnosis of benign breast tumors or breast cancer, as needed.

**Figure 2:**
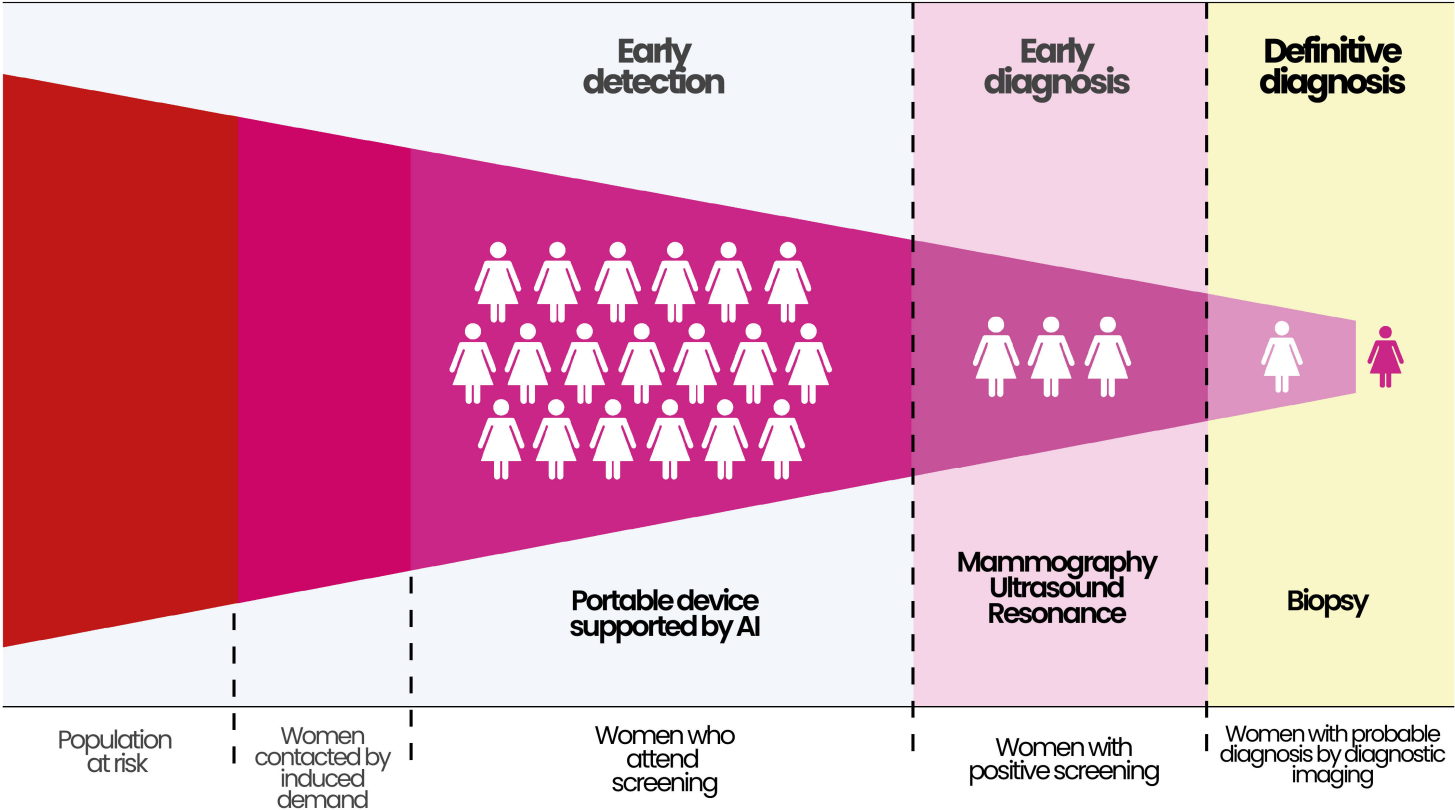
*Cuídalas* Program screening pathway: from population at risk to definitive diagnosis, integrating AI-supported portable screening, diagnostic imaging, and biopsy. AI, Artificial Intelligence; Source, authors.

The approach focused on ensuring timely access to care aims to ensure the continuity of timely services, facilitating early diagnosis, specialized evaluation, and prompt treatment in cases of breast cancer (**Figure 2**). Additionally, ongoing communication and support are maintained to ensure access to rehabilitation services and palliative care according to the needs of the women.

Between March 2023 and September 2024, the *Cuídalas* Program operated across multiple regions of Colombia in collaboration with several national health insurance providers, reaching 346,534 women registered within participating networks. As part of its community-based induced-demand strategy, 54,970 women accessed screening services during this period. Aggregated program records indicate that 6.22% of screened women presented findings requiring further clinical assessment.

Through its structured referral and navigation pathways, the program facilitated subsequent medical evaluation by general practitioners or gynecologists in 75.65% of cases with suspicious findings. Diagnostic imaging was performed according to clinical indication, including 151 mammograms and 1,319 breast ultrasounds. Across these assessments, 533 examinations were categorized as Breast Imaging Reporting and Data System (BI-RADS) 3, 4, 5, or 6.

Within the continuum of care activated by the program, 15 breast cancer diagnoses were confirmed through standard clinical pathways (**Table 1**). At the time of reporting, 12 women were undergoing treatment, two had completed therapy without evidence of disease, and one death had been documented. These figures reflect aggregated operational outputs of routine service delivery rather than outcomes derived from a designed clinical investigation.

**Table 1:** Preliminary operational results of the *Cuídalas* program (March 2023 - September 2024).

Indicator	Frequency	Percentage	Description
Total women enrolled	346,534	100	Women affiliated with national health insurance companies
Women screened	54,970	15.90	Completed AI-supported screening using the *iBreastExam™* device
Positive findings	3,421	6.20	Women with abnormal results requiring follow-up
Women with follow-up by general practitioner/gynecologist	2,588	75.60	Received clinical evaluation after positive screening
Mammograms performed	151	–	Conducted as confirmatory diagnostic imaging
Breast ultrasounds performed	1,319	–	Complementary imaging in follow-up process
Abnormal imaging results (BI-RADS 3-6)	533	–	Includes both mammograms and ultrasounds
Confirmed breast cancer cases	15	–	Diagnoses confirmed by histopathology

Breast Imaging Reporting and Data System (BI-RADS).

In practice, all initial screenings were performed by nurses or nursing assistants. These healthcare professionals underwent a standardized one-week training program prior to deployment, which included modules on breast anatomy and pathology, patient communication, cultural competence, device handling and calibration, breast visual inspection techniques, and clinical protocols for triaging patients based on screening results.

The implementation process was not without challenges. Some nurses initially experienced difficulties in using the AI-powered device due to limited prior exposure to digital health technologies. Others faced barriers related to community engagement, particularly in areas with low health literacy or skepticism toward new technologies. These challenges were mitigated through on-site supervision, continuous technical support, and culturally tailored health education materials co-developed with nursing teams. In addition to performing screenings, nurses were pivotal in counseling patients, reinforcing adherence, and navigating referrals, thereby strengthening the continuity and comprehensiveness of the care pathway.

This program aims to reduce mortality from late-stage breast cancer diagnoses, decrease potentially avoidable early mortality from breast cancer, lower medical costs, reduce the need for aggressive treatments, and improve the quality of life for women with breast cancer.

Beyond their operational role, nurses in the *Cuídalas* Program served as cultural mediators between technology and community. Through their direct contact with women in diverse geographic and social contexts, they built trust, translated complex information into accessible messages, and tailored educational interventions for populations with limited health literacy. This culturally sensitive approach was essential to counteract fear, misinformation, and skepticism toward screening technologies.

Training modules developed for the program were designed not only to ensure technical proficiency with the AI-supported device but also to strengthen nurses’ communication, leadership, and community engagement skills. This capacity-building model promotes local sustainability by empowering nursing teams to independently conduct, monitor, and expand the program’s reach.

These preliminary results suggest that the *Cuídalas* Program is both feasible and acceptable within diverse community settings. The integration of artificial intelligence–supported portable screening, combined with nursing-led outreach, enabled implementation in areas with limited infrastructure and high geographic dispersion. High follow-up rates among women with positive findings indicate that the model successfully linked community-based screening with the formal healthcare system, demonstrating operational coherence and community receptivity.

Several key facilitators contributed to the program’s success during its initial phase. Effective communication strategies, rapid response to logistical needs, and continuous technical support were essential in maintaining workflow efficiency. Most importantly, the leadership and engagement of nursing teams fostered trust, improved adherence, and enhanced understanding of screening processes among participants. These elements underscore nursing professionals as central drivers of innovation and implementation in community-based cancer prevention.

Despite encouraging outcomes, several barriers emerged during real-world implementation. Device calibration and connectivity occasionally posed technical challenges, particularly in remote areas with unstable infrastructure. Cultural mistrust and low digital literacy also limited initial participation in some communities. Continuous training, on-site supervision, and the use of culturally tailored health education materials were critical to overcoming these barriers, reinforcing the importance of sustained human engagement alongside technology deployment.

If scaled nationally, this community-engaged, AI-assisted screening model could contribute substantially to bridging health equity gaps in breast cancer prevention. The *Cuídalas* Program illustrates how combining advanced technology with local nursing capacity can promote early detection, cost efficiency, and sustainability in low-resource settings. Future evaluations should focus on long-term clinical outcomes, cost-effectiveness, and policy integration to strengthen evidence-based decision-making for equitable breast health interventions.

## 4 Portable devices supported by artificial intelligence: tools with the potential to overcome geographic and cultural barriers led by nursing practice and multidisciplinary teams

One of the benefits of the *Cuídalas* Program is the use of portable devices supported by artificial intelligence. In this case, the *iBreastExam™* was used. This is a handheld, battery-operated device equipped with a set of dynamic pressure sensors. This device has 648 dynamic coplanar capacitive sensors that accurately assess and identify differences in tissue elasticity between hard, stiff breast tumors and normal breast tissue in real time. The patented tactile sensor technology is a novel, quantitative elastic module that can measure tissue compression and stiffness by touching the skin surface from top to bottom (10).

These sensors are designed to effectively identify distinctions between hard or rigid areas and healthy breast tissue. The collected data are represented on a 3D surface map that illustrates the stiffness profile. Results are transmitted wirelessly to a tablet, which subsequently uploads them to a secure cloud (10). The device generates a color map that does not necessitate interpretation by a physician, where red signifies areas that require further examination. The *iBreastExam™* device has undergone upgrades (from versions W00009/W00010 to W00008) to enhance calibration simplicity, with subanalysis conducted on the data collected before and after this upgrade (10).

Compared to a manual clinical breast exam, the *iBreastExam™* has demonstrated up to 86% sensitivity for any positive findings, up to 94% specificity, and negative and positive predictive values of 98% and 66%, respectively (11). Evidence has supported that a screening test refers to the application of a medical procedure or test to individuals who do not yet exhibit symptoms of a specific disease, in order to determine their likelihood of developing it (36, 37). Recent device versions incorporate simplified calibration and autocalibration features, enabling standardized use by trained healthcare personnel and facilitating deployment in outreach programs (11).

The screening procedure itself does not diagnose the disease. Individuals who receive a positive result from the screening test will require further evaluation through diagnostic tests or procedures. This eliminates the requirement for a screening test to perform at the same level as a diagnostic tool, which provides a probable or definitive diagnosis of a disease (such as mammography or ultrasound). This is not one of the qualities of screening tools (36–38). Therefore, in line with the performance parameters for this type of test, it is expected to be highly sensitive, but not specific.

**Figure 3:**
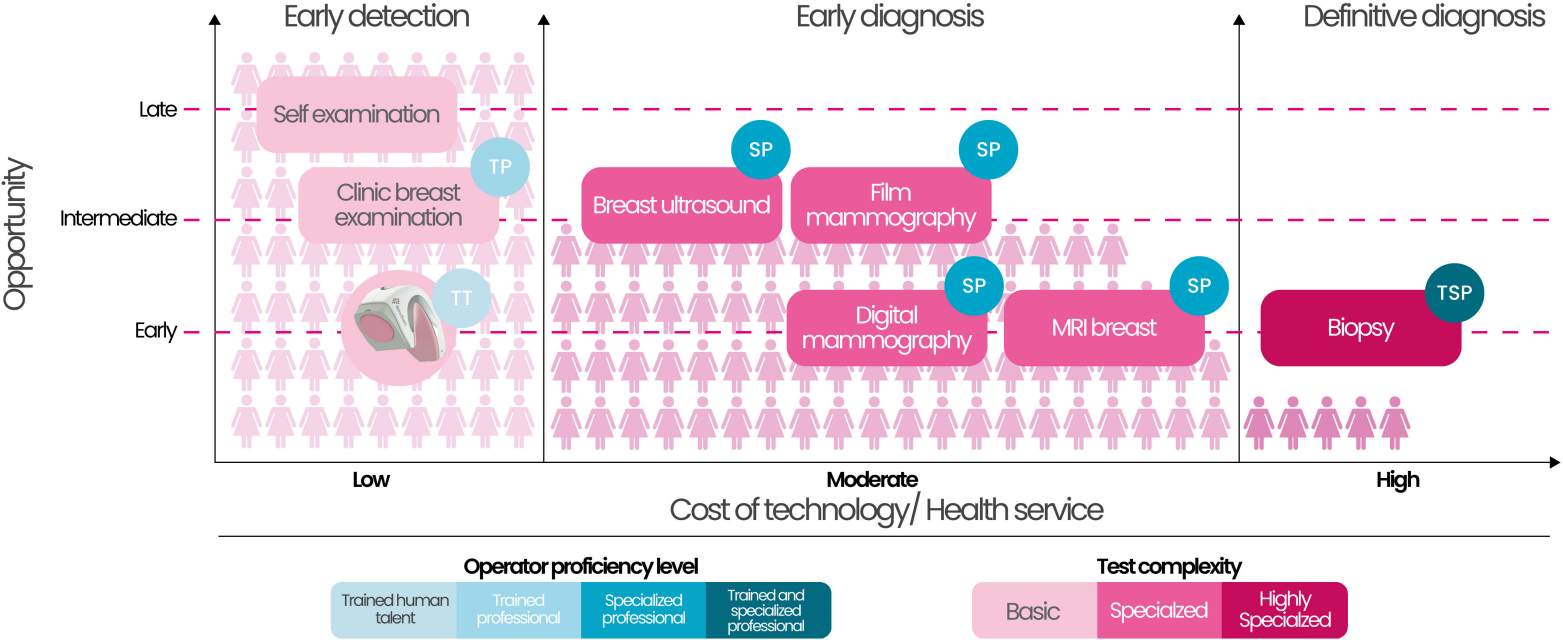
Comparative analysis of breast cancer screening modalities by opportunity, cost, test complexity, and human resource requirements, highlighting the scalability of the *Cuídalas* Program’s AI-supported approach. The *Cuidalas* program employs a low-cost, portable device supported by artificial intelligence (in this case, the *iBreastExam™*), which can be operated by trained personnel who are not necessarily healthcare professionals, enabling large-scale screening for early detection of breast abnormalities in low-resource settings. TP, Training Phase; TT, Technical Testing; SP, Screening Phase; TSP, Treatment and Surveillance Phase. Source, authors.

In settings characterized by complex socioeconomic, technological, cultural, and healthcare contexts, such as in Colombia, where international scientific discourse has highlighted and acknowledged the limitations of resources, the shortage of highly trained human talent, and cultural and geographical barriers, among others, as well as the eventual negative impact and inability to provide screening coverage and timely access for women at risk, the emergence of a screening tool that can detect the highest number of individuals with positive results (high sensitivity) would be of great value (39). This ensures a favorable balance between costs, opportunity, human talent required to implement the screening and accessibility to the test (**Figure 3**).

In Colombia, many women in rural or low-income areas do not have access to breast cancer screening tests, whether through clinical breast exams or diagnostic imaging, which creates an unfavorable scenario for vulnerable populations. This issue has been emphasized as a priority in the review of breast cancer care and outcomes in Latin America, where it is noted that substantial inequities exist in Colombia regarding access to screening programs for these vulnerable populations. These inequities persist, despite screening guidelines and recommendations established over a decade ago, which fail to consider these sociocultural barriers (17). As a result, it is inferred that, in these settings, breast cancer is often detected in advanced stages, with devastating consequences for both patients and the healthcare system.

Advanced nursing practice, combining community-health expertise, clinical technology skills, and health-education proficiency, forms the integrative backbone of the *Cuídalas* Program. Community nurses steer the demand-induction phase by rapidly assessing breast-cancer literacy and tailoring educational messages to local dialects and cultural sensitivities. During the organized screening phase, nurse managers act as digital gatekeepers between the client and the AI triage engine, ensuring high-quality image acquisition, enforcing dual-reading protocols, and significantly reducing false positives. This unique blend of clinical decision-making, culturally competent communication, and technological fluency positions nursing professionals as natural leaders of initiatives that deploy artificial intelligence-enabled portable devices in low-resource settings.

Despite the demonstrated sensitivity and specificity of the *iBreastExam™* device, some operational limitations must be considered in real-world deployment. First, although recent device upgrades have simplified calibration procedures, ensuring consistent and accurate performance still requires routine quality checks. Second, the device’s effectiveness depends on proper user training; while the learning curve is not steep, inexperienced operators may introduce variability. Third, as with any highly sensitive screening tool, there is a risk of false-positive results, which may lead to unnecessary referrals, anxiety, or additional tests. To mitigate these issues, the *Cuídalas* Program has implemented a structured training protocol and a dual-reading system, ensuring that results are reviewed by trained nurse supervisors or physicians prior to initiating referral pathways. These measures aim to preserve the program’s scalability while minimizing unintended consequences of technology deployment in underserved communities.

Then, portable devices supported by AI emerge as an alternative screening tool for assessing breast health, suitable for use in low-resource settings. It provides a tangible result that facilitates the monitoring and inclusion of women in a comprehensive care pathway for the early detection of breast cancer, differing from the subjective perceptions of the operator during a clinical breast examination. Therefore, it serves as a complementary tool, with the potential to help address a global health issue and public health challenge.

The real-world implementation of portable, AI-supported devices in community contexts presented several challenges beyond technical performance. A proportion of false-positive findings generated understandable concern and anxiety among women, especially in communities with limited prior exposure to screening programs. To address this, nursing staff received additional training in empathetic communication and post-screening counseling, ensuring reassurance and clear explanations about follow-up procedures.

Furthermore, maintaining quality and consistency in device use required sustained investment in supervision and refresher training, positioning continuous capacity-building as a core component of the *Cuídalas* model. Finally, the long-term success of such innovations depends on community trust in technology, which requires sustained engagement and transparent communication strategies. Early engagement strategies, transparency in communication, and active participation of local nursing leaders proved essential to foster acceptance of AI-supported tools and mitigate initial skepticism.

## 5 Future perspectives

It is important to note that the findings reported are preliminary and reflect operational performance during the initial implementation phase. The purpose of this analysis is to document feasibility, community engagement, and nursing leadership within an AI-supported framework, providing the foundation for future evaluative and impact studies.

The *Cuídalas* Program is currently conducting several studies using mixed-method research designs to describe the key factors associated with the success or failure of specific primary care strategies at each stage, from initial contact with at-risk women requiring breast cancer screening, to the follow-up and rehabilitation of women after treatments for breast cancer or other breast neoplasms. Although not thoroughly explored, there is a significant knowledge gap regarding the phenomenological characteristics of the health and illness processes of breast cancer in vulnerable regions (40), particularly in countries with considerable cultural, ethnic, and racial diversity, which hinders the implementation of traditional care programs (17, 18, 40).

Challenges such as illiteracy, multidimensional and extreme poverty, health education in indigenous communities, and the lack of recognition of women as active and participatory members of society (still prevalent in some communities) must be addressed through evidence-based research approaches (41). These approaches require the collection of specific primary data for designing relevant studies, enabling evidence-based decision-making to improve health outcomes and development indicators related to breast cancer and its disease burden in low- and middle-income countries (42).

Preliminary results demonstrate the operational feasibility and acceptability of implementing an AI-supported, nursing-led screening model in diverse community settings. These findings reflect real-world observations based on the initial deployment phase.

In contrast, the potential for large-scale implementation remains a future perspective, contingent on ongoing evaluations of effectiveness, sustainability, and cost-efficiency. If scaled nationally, the *Cuídalas* model could contribute to improving early detection coverage and strengthening nursing capacity in underserved areas.

The objective of this report is not to demonstrate the clinical effectiveness of the *Cuídalas* Program, but rather to present its structural design, implementation strategy, and operational reach. Comprehensive statistical analyses evaluating the program’s impact on breast cancer outcomes are currently underway and will be presented in future publications.

Subsequent analyses will include stratified sociodemographic and clinical data to better understand population-level patterns, health equity gaps, and screening performance across different regions and age groups.

## 6 Conclusions

The *Cuídalas* Program represents a nursing-led, community-based primary care initiative supported by AI and portable digital tools. Preliminary results confirm its feasibility and operational effectiveness in increasing access to breast cancer screening in low-resource contexts. These early findings reflect the capacity of nursing teams to integrate technology with community engagement, bridging equity gaps in preventive care.

Future stages of the program will focus on evaluating its impact on clinical outcomes, cost-effectiveness, and scalability across different regions. By emphasizing capacity building, cultural sensitivity, and trust, *Cuídalas* positions nursing professionals at the center of a sustainable, AI-assisted model for early cancer detection in underserved populations.

## References

[1] World Health Organization . Breast cancer. Available online at: https://www.who.int/news-room/fact-sheets/detail/breast-cancer (Accessed December 22, 2024).

[2] International Agency for Research on Cancer . Breast. Available online at: https://gco.iarc.who.int/media/globocan/factsheets/cancers/20-breast-fact-sheet.pdf (Accessed December 22, 2024).

[3] Xu Y Gong M Wang Y Yang Y Liu S Zeng Q . Global trends and forecasts of breast cancer incidence and deaths. Sci Data. (2023) 10:334. doi: 10.1038/s41597-023-02253-5. PMID: 37244901 PMC10224917

[4] Trapani D Ginsburg O Fadelu T Lin NU Hassett M Ilbawi AM . Global challenges and policy solutions in breast cancer control. Cancer Treat Rev. (2022) 104:102339. doi: 10.1016/j.ctrv.2022.102339. PMID: 35074727

[5] Andrade AV Lucena CÊM Santos DCD Pessoa EC Mansani FP Andrade FEM . Challenges of breast cancer screening. Rev Bras Ginecol Obstet. (2023) 45:551–4. doi: 10.1055/s-0043-1775931. PMID: 37846189 PMC10579917

[6] Schliemann D Hoe WMK Mohan D Allotey P Reidpath DD Tan MM . Challenges and opportunities for breast cancer early detection among rural dwelling women in Segamat District, Malaysia: A qualitative study. PloS One. (2022) 17:e0267308. doi: 10.1371/journal.pone.0267308. PMID: 35594267 PMC9122189

[7] Esserman L the WISDOM Study and Athena Investigators . The WISDOM Study: breaking the deadlock in the breast cancer screening debate. NPJ Breast Cancer. (2017) 3:34. doi: 10.1038/s41523-017-0035-5. PMID: 28944288 PMC5597574

[8] Sayed S Ngugi AK Nwosu N Mutebi MC Ochieng P Mwenda AS . Training health workers in clinical breast examination for early detection of breast cancer in low- and middle-income countries. Cochrane Database Syst Rev. (2023) 4:CD012515. doi: 10.1002/14651858.cd012515. PMID: 37070783 PMC10122521

[9] Marinovich ML Wylie E Lotter W Lund H Waddell A Madeley C . Artificial intelligence (AI) for breast cancer screening: BreastScreen population-based cohort study of cancer detection. EBioMedicine. (2023) 90:104498. doi: 10.1016/j.ebiom.2023.104498. PMID: 36863255 PMC9996220

[10] Mango VL Olasehinde O Omisore AD Wuraola FO Famurewa OC Sevilimedu V . The iBreastExam versus clinical breast examination for breast evaluation in high risk and symptomatic Nigerian women: a prospective study. Lancet Glob Health. (2022) 10:e555–63. doi: 10.1016/s2214-109x(22)00030-4. PMID: 35303464 PMC9102465

[11] Bhimani F Zhang J Shah L McEvoy M Gupta A Pastoriza J . Can the clinical utility of iBreastExam, a novel device, aid in optimizing breast cancer diagnosis? A systematic review. JCO Glob Oncol. (2023) 9:e2300149. doi: 10.1200/go.23.00149. PMID: 38085036 PMC10846782

[12] Hinzey A Gaudier-Diaz MM Lustberg MB DeVries AC . Breast cancer and social environment: getting by with a little help from our friends. Breast Cancer Res. (2016) 18:54. doi: 10.1186/s13058-016-0700-x. PMID: 27225892 PMC4881170

[13] Coughlin SS . Social determinants of breast cancer risk, stage, and survival. Breast Cancer Res Treat. (2019) 177:537–48. doi: 10.1007/s10549-019-05340-7. PMID: 31270761

[14] Shulman LN Willett W Sievers A Knaul FM . Breast cancer in developing countries: opportunities for improved survival. J Oncol. (2010) 2010:595167. doi: 10.1155/2010/595167. PMID: 21253541 PMC3021855

[15] Kantelhardt EJ Assefa M McCormack V Cubasch H Jemal A Pace LE . Expert discussion: breast cancer in low-resource countries. Breast Care (Basel). (2020) 15:310–3. doi: 10.1159/000508693. PMID: 32774226 PMC7383243

[16] Francies FZ Hull R Khanyile R Dlamini Z . Breast cancer in low-middle income countries: abnormality in splicing and lack of targeted treatment options. Am J Cancer Res. (2020) 10:1568–91 (Accessed December 22, 2024). PMC7269781 32509398

[17] Pan American Health Organization . A review of breast cancer care and outcomes in Latin America. Available online at: https://www.paho.org/es/file/32279/download?token=fvYVv9hn (Accessed December 22, 2024).

[18] World Health Organization . Breast Cancer Outcomes in Sub-Saharan Africa. Available online at: https://www.iarc.who.int/wp-content/uploads/2021/03/IARC_Evidence_Summary_Brief_1.pdf (Accessed December 22, 2024).

[19] World Health Organization . The Global Breast Cancer Initiative: Empowering women, building capacity, providing care for all. Available online at: https://www.who.int/initiatives/global-breast-cancer-initiative/breast-cancer-inequities#:~:text=Several%2C%20high%2Dincome%20countries%20have,%2D%20and%20middle%2Dincome%20countries (Accessed December 22, 2024).

[20] El Saghir NS Adebamowo CA Anderson BO Carlson RW Bird PA Corbex M . Breast cancer management in low resource countries (LRCs): consensus statement from the Breast Health Global Initiative. Breast. (2011) 20:S3–11. doi: 10.1016/j.breast.2011.02.006. PMID: 21392996

[21] World Bank Group . Gini index. Available online at: https://data.worldbank.org/indicator/SI.POV.GINI (Accessed December 22, 2024).

[22] World Bank Group . Multidimensional poverty headcount ratio (World Bank) (% of population). Available online at: https://data.worldbank.org/indicator/SI.POV.MPWB?end=2023&start=2008&view=chart (Accessed December 22, 2024).

[23] Instituto Nacional de Cancerología de Colombia . Epidemiological Bulletin No. 10 (2017). Available online at: https://www.cancer.gov.co/recursos_user/files/libros/archivos/B (Accessed December 22, 2024).

[24] Colombia High-Cost Account . World Breast Cancer Awareness Day 2023 (2017). Available online at: https://cuentadealtocosto.org/cancer/dia-mundial-de-la-lucha-contra-el-cancer-de-mama-2023/ (Accessed December 22, 2024).

[25] Weisner C Díaz S Sánchez O Puerto D Bravo LE Murillo R . Evidence-based policies: the case of breast cancer control in Colombia. Rev Colomb Cancerol. (2020) 24:98–107.

[26] Castiblanco C . Health professionals wanted. Conexxión. (2024) 28:18–20.

[27] National Administrative Department of Statistics of Colombia . Monetary poverty in Colombia according to social class. Available online at: https://www.dane.gov.co/files/operaciones/PM/cp-PMClaseSociales-2023.pdf (Accessed December 22, 2024).

[28] Mantilla-Duran WA Franco Millan SXXX Bravo MA Cervera SB Osorio AM Caicedo Mallarino JJ . 296P Impact of screening strategies on the cost of breast cancer care in Colombia. ESMO Open. (2025) 10:104851. doi: 10.1016/j.esmoop.2025.104851. PMID: 41859675

[29] Duarte C Salazar A Strasser-Weippl K de Vries E Wiesner C Arango-Gutiérrez A . Breast cancer in Colombia: a growing challenge for the healthcare system. Breast Cancer Res Treat. (2021) 186:15–24. doi: 10.1007/s10549-020-06091-6. PMID: 33611666

[30] Arboleda W Murillo R Piñeros M Perry F Díaz S Salguero E . Cobertura de examen clínico y mamografía de tamización para cáncer de mama en mujeres bogotanas. Rev Colombiana Cancerol. (2009) 13:69–76.

[31] International Agency for Research on Cancer . Country Fact Sheet: Colombia. Available online at: https://canscreen5.iarc.fr/?page=countryfactsheet&q=COL (Accessed December 22, 2024).

[32] DeBoer RJ Fadelu TA Shulman LN Van Loon K . Applying lessons learned from low-resource settings to prioritize cancer care in a pandemic. JAMA Oncol. (2020) 6:1429–33. doi: 10.1001/jamaoncol.2020.2976. PMID: 32761149

[33] Instituto Nacional de Cancerología de Colombia . Manual for the Early Detection of Breast Cancer. Available online at: https://www.cancer.gov.co/recursos_user/files/libros/archivos/,Manual#:~:text=A%20diferencia%20de%20la%20mamograf%C3%ADa,los%2050%20y%2069%20a%C3%B1os (Accessed December 22, 2024).

[34] Instituto Nacional de Cancerología de Colombia . Clinical practice guideline (CPG) for early detection, comprehensive treatment, follow-up and rehabilitation of breast cancer. Available online at: https://www.minsalud.gov.co/sites/rid/1/Gu%C3%ADa%20de%20Pr%C3%A1ctica%20Cl%C3%ADnica%20%20de%20Cancer%20de%20Mama%20versi%C3%B3n%20completa.pdf (Accessed December 22, 2024).

[35] Pan American Health Organization . WHO Position Paper on Mammography Screening: Summary of Recommendations. Available online at: https://www3.paho.org/hq/dmdocuments/2015/WHO-ENG-Mammography-Factsheet.pdf (Accessed December 22, 2024).

[36] Institute for Quality and Efficiency in Health Care (IQWiG) . In brief: Benefits and risks of screening tests. Cologne, Germany: Institute for Quality and Efficiency in Health Care (IQWiG (2019). Available online at: https://www.ncbi.nlm.nih.gov/books/NBK279418/ (Accessed December 22, 2024).

[37] Department of Health of New York State . Disease Screening - Statistics Teaching Tools. Available online at: https://www.health.ny.gov/diseases/chronic/discreen.htm#:~:text=The%20more%20sensitive%20a%20test,greater%20the%20positive%20predictive%20value (Accessed December 22, 2024).

[38] Narayan AK Miles RC Woods RW Spalluto LB Burnside ES . Methodological considerations in evaluating breast cancer screening studies. J Breast Imaging. (2024), 6(6):wbae038. doi: 10.1093/jbi/wbae038. PMID: 39096512

[39] Pinto JA Pinillos L Villarreal-Garza C Morante Z Villarán MV Mejía G . Barriers in Latin America for the management of locally advanced breast cancer. Ecancermedicalscience. (2019) 13:897. doi: 10.3332/ecancer.2019.897. PMID: 30792814 PMC6372299

[40] Armour-Burton T Etland C . Black feminist thought: A paradigm to examine breast cancer disparities. Nurs Res. (2020) 69:272–9. 10.1097/NNR.0000000000000426 32040048

[41] Yedjou CG Sims JN Miele L Noubissi F Lowe L Fonseca DD . Health and racial disparity in breast cancer. Adv Exp Med Biol. (2019) 1152:31–49. doi: 10.1007/978-3-030-20301-6_3. PMID: 31456178 PMC6941147

[42] Picón-Jaimes YA . Innovation and digital transformation in health education: opportunities to drive technological development in the training of future professionals. Inge CuC. (2024) 20:99–105. doi: 10.17981/ingecuc.20.2.2024.10

